# The Prevention of Bleeding during Long-Term Oral Anticoagulant Therapy

**Published:** 1972-01

**Authors:** R. D. Eastham

**Affiliations:** Consultant Pathologist to the Frenchay Hospital Group


### The Prevention of Bleeding During Long-Term Oral Anticoagulant Therapy

**R. D. Eastham, M.D., M.R.C.P., F.R.C.Path., D.C.P., Dipl.Path.**

Consultant Pathologist to the Frenchay Hospital Group

The results obtained in the Anticoagulant Outpatient Clinic during the five-year period January 1967—December 1971 have been examined to see whether there has been any improvement in the standard of anticoagulant control, with reduction in the number of haemorrhagic episodes. Unfortunately, these results are disappointing, since apparently avoidable haemorrhages have occurred, and these are now reported together with some suggestions which it is hoped may lead to some improvement in the control of oral anticoagulant therapy in future. The importance of thorough clinical supervision and frequent regular blood tests during the first few weeks of such treatment, along with the need to avoid drugs known either to potentiate or reduce oral anticoagulant action is stressed, since any haemorrhage during anticoagulant therapy is potentially dangerous.

### Methods and Materials

4,250 paired prothrombin ratios and plasma activated partial thromboplastin clotting times from 301 patients were examined during 1967-1971. Warfarin (or phenindione in a few cases) was given to maintain the plasma activated partial thromboplastin clotting time within a therapeutic range of 50-70 seconds (Eastham, 1968). The patients included 88 patients treated following myocardial infarction, 153 patients treated following venous thrombosis, 32 patients with mitral valve disease treated to prevent embolic attacks, and 28 patients treated following arterial thromboses other than coronary artery thrombosis.

### Results

During the five years, 1967-71, 301 patients attended the Anticoagulant Outpatient Clinic following discharge from the hospital wards, anticoagulant therapy having been initiated while they were In-patients. 103 haemorrhagic episodes were recorded in the Outpatient Clinic during a total of 3,445 man/months of anticoagulant treatment. When the timing of these episodes was examined, it was found that 32 haemorrhages had occurred during the first four weeks since anticoagulant therapy had been started in the hospital wards, a further 15 haemorrhagic episodes had occurred during the second four weeks of anticoagulant treatment, and a further three haemorrhages had occurred during the third month of treatment. Thus nearly half of the total bleeding episodes had occurred soon after anticoagulant treatment had been given.

Severity of haemorrhage varied from minor to severe, with 37 severe haemorrhages which included one fatal intracranial haemorrhage, and six gastrointestinal haemorrhages necessitating either cessation of anticoagulant treatment or admission to hospital. The possible causes of haemorrhage were carefully examined and it was found that a total of 38 episodes could be explained by causes other than anticoagulant overdosage alone (Table 1). The sites of haemorrhages are shown in Table (2).

**TABLE 1**

**CONDITIONS PROBABLY ASSOCIATED WITH HAEMORRHAGE**

| Agent causing haemorrhage | Occurring before 12 wks. | After 12 wks. | Total |
|---|---|---|---|
| Phenylbutazone ... | 7 | 1 | 8 |
| Anticonvulsants ... | 3 | 2 | 5 |
| Unreliable patient (dosage) ... ... | 2 | 0 | 2 |
| Tranquillizers etc. ... | 1 | 4 | 5 |
| Antibiotics (broad-spectrum) ... | 0 | 7 | 7 |
| Clofibrate ... ... | 0 | 2 | 2 |
| Congestive cardiac failure ... ... | 0 | 3 | 3 |
| Thrombocytopenia ... | 0 | 3 | 3 |
| Steroid therapy (more than 15 mg prednisolone/ day) ... ... ... | 1 | 1 | 2 |
| Bladder papilloma ... | 0 | 1 | 1 |
| Total known causes | 14 | 24 | 38 |
| Total with no other treatment than oral anticoagulants | 36 | 29 | 65 |
| | 50 | 53 | 103 |

**TABLE 2**

**SITES OF BLEEDING IN 103 EPISODES**

| | |
|---|---|
| Subcutaneous bruising ... ... ... ... ... | 50 |
| Haematuria ... ... ... ... ... | 35 |
| Subconjunctival haemorrhage ... ... ... | 9 |
| Gastrointestinal bleeding ... ... ... ... | 6 |
| Epistaxis ... ... ... ... ... ... | 3 |
| Retroperitoneal haemorrhage ... ... ... | 2 |
| Bleeding gums ... ... ... ... ... | 2 |
| Intracranial haemorrhage (fatal) ... ... ... | 1 |
| | 108 |

(1 case Bruises + epistaxis
2 cases Bruises + haematuria
1 case melaena + subconjunctival haemorrhage
1 bruises + subconjunctival haemorrhage)

**Discussion**

Any haemorrhage is a potentially serious complication of oral anticoagulant therapy: for example, during high-dosage treatment with oral anticoagulants, 80 haemorrhages occurred during 5,101 patient/months of treatment of 195 patients, and of these, 16 were serious (Medical Research Council, 1964). Straub (1970) reported 13 deaths from haemorrhage during oral anticoagulant treatment of 70 patients, while in another series, 10 patients developed intracranial haemorrhages during two and a half years of observation of patients treated with anticoagulants (Hazard et al, 1967), and these latter workers concluded that haemorrhages had occurred as a result of three main causes, namely, the presence of a medical contraindication to anticoagulant treatment, insufficient clinical supervision of patients, or insufficient laboratory control of anticoagulant dosage. Serradimigni and his co-workers (1969) found an incidence of 76 haemorrhagic episodes in 507 patients treated during a five-year period of observation. They found that of all patients on whom laboratory tests were carried out at the time of the haemorrhage, 17 had prothrombin times within the therapeutic range, and similar findings were reported by Eastham (1968). On the other hand, 31 patients had prolonged prothrombin times indicating a potential haemorrhage risk, and they suggested that these latter cases had bled because of errors of dosage by patients, possible changes of diet with variations in vitamin K intake, drugs known to alter warfarin action or medical or surgical conditions known to be associated with haemorrhage and unmasked by warfarin.

It is known that following oral warfarin, the average half-time of disappearance of the drug from the blood is 44 hours (range 15-56 hours) (O'Reilly et al, 1963), and the plasma concentrations of Factors II, VII, IX, and X, are related to the rate of elimination of warfarin, if the dose is such that synthesis of these plasma factors is not completely suppressed (Nagashima et al, 1969). Zieve and Solomon (1969) have shown that there is considerable variation in normal people of the effect of vitamin K when it is absorbed after warfarin has been given, and this indicates that different individuals will require different doses of warfarin to produce the same incomplete suppression of synthesis of vitamin K-dependent plasma clotting factors, necessary for safe yet thorough anticoagulant therapy.

Ideally, it should be possible so to adjust the dose of oral anticoagulants by means of the results from regular blood tests, so that haemorrhagic complications do not occur, and the plasma clotting factors maintained within the optimum therapeutic range to prevent further thromboses. With the knowledge that certain drugs potentiate and other drugs depress the action of oral anticoagulant drugs, iatrogenic haemorrhages should also be avoidable (Hamblin, 1970, Koch-Weser, & Seller, 1971).

In the present series of 103 haemorrhagic episodes during five years of outpatient treatment, more than one third of the haemorrhages appear to have occurred directly as a result of administration of drugs which are known to interfere in some way with warfarin metabolism, or as a result of medical conditions known to cause haemorrhage when warfarin is given. No simple explanation for the remaining two thirds of the haemorrhages was obtained, but a disturbing new factor became apparent when the time of each haemorrhagic episode was examined in relation to the duration of oral anticoagulant therapy. Nearly one third of the episodes occurred during the first month, and 46% of the episodes occurred during the first two months.

It is therefore suggested that when a patient is started on oral anticoagulant therapy (with or without concurrent heparin therapy during the first four days), warfarin should be given at the rate of 10-15 mg per day, depending on the patient's weight. An initial loading dose should be avoided, since this produces marked reduction in plasma Factor VII with prolongation of the prothrombin time within 24-48 hours, with only minimal effects on plasma Factors II, IX, and X, and hence with only minimal effect on the activated partial thromboplastin time and intrinsic coagulation (Deykin, 1970). The initial depression of Factors IX and X which follows continuous daily administration of 10-15 mg warfarin is as rapid and as marked as after a large initial dose followed by smaller daily doses (O'Reilly & Aggeler, 1968). After the first 4-5 days of treatment with warfarin, the prothrombin ratio and the activated partial thromboplastin clotting time reflect warfarin action either as depression of Factors II, VII, and X, or of Factors II, IX, and X respectively. In this context the prothrombin test is probably as reliable as the activated partial thromboplastin time in most cases as long as the daily dose of warfarin is not altered.

The anticoagulant treatment in the hospital ward should be regular and frequent, with administration of warfarin at the same time each day; since the half-life of Factor VII in the plasma is only 4-5 hours, warfarin dosage should be 12-hourly when possible, and daily changes of warfarin dosage should be avoided. Patients discharged to continue on long-term anticoagulant therapy as outpatients should have appointments to attend the Anticoagulant Outpatient Clinic for clotting tests as soon as possible after leaving the hospital ward, and certainly within one week of discharge. These patients should carry a list of drugs which should either be avoided altogether or only prescribed if the prescription is notified to the Anticoagulant Clinic. These same lists should be displayed in the hospital wards. At the time of discharge from hospital, patients should be instructed that should they notice unexplained bruising or visible haematuria, they should not take any further doses of warfarin until they had sought the advice either of their General Practitioner or at the Hospital Outpatient Department. Attendance at the Outpatient Clinic should be weekly for the first few weeks until dose stability with satisfactory depression of plasma clotting factors has been obtained. In this way, it should be possible to reduce the number of bleeding episodes due to inadequately controlled warfarin therapy.

**References**

1. Deykin, D. (1970). Warfarin Therapy (Part One). New England Journal of Medicine. 283, 691.

2. Eastham, R. D. (1968). Improved Control of long-term Anticoagulant Therapy. British Medical Journal. 2, 337.

3. Hamblin, T. J. (1970). The Problems of Anticoagulant Control. 1. Drug Interactions. Bristol Medico-Chirurgical Journal. 85, 101.

4. Hazard, J., Mignot, B., Gaubler, J. P., Domart, A. (1967). A propos de dix hemorragies intracraniennes survenues au cours de traitements anticoagulants. Semaine des Hopitaux de Paris. 43, 2849.

5. Koch-Weser, J., and Sellers, E. M. (1971). Drug interactions with Coumarin anticoagulants. New England Journal of Medicine. 285, 487 and 547.

6. Nagashima, R., O'Reilly, R. A., Levy, G. (1969). Kinetics of pharmacologic effects in man: the anticoagulant action of warfarin. Clinical Pharmacology and Therapeutics. 10, 22.

7. O'Reilly, R. A., and Aggeler, P. M. (1968). Studies on coumarin anticoagulant drugs: initiation of warfarin therapy without a loading dose. Circulation. 38, 169.

8. O'Reilly, R. A., Aggeler, P. M., Leong, L. S. (1963). Studies on coumarin anticoagulant drugs: pharmacodynamics of warfarin in man. Journal of Clinical Investigation. 42, 1542.

9. Second Report of the Working Party on Anticoagulant Therapy in Coronary Thrombosis to the Medical Research Council (1964). An Assessment of long-term Anticoagulant Administration after cardiac infarction. British Medical Journal. 2, 837.

10. Serradimigni, A., Arnoux, E., Raybaud, M., Giraud, M., Pinas, E., Audier, M. (1969). Les hemorragies des anticoagulants. Marseille Medical. 106, 1041.

11. Straub, P. W. (1970). Indikationen und Gefahren der Antikoagulantientherapie. Schweizische Medizinische Woschenschrift. 100, 686.

12. Zieve, P. D., and Solomon, H. M. (1969). Variations in the response of human beings to vitamin K. Journal of Laboratory and Clinical Medicine. 73, 103.

